# Inhibition of integrin subunit alpha 11 restrains gastric cancer progression through phosphatidylinositol 3-kinase/Akt pathway

**DOI:** 10.1080/21655979.2021.2006551

**Published:** 2021-12-19

**Authors:** Haijun Zhang, Lin Zhang, Ming Lu

**Affiliations:** a Second Department of General Surgery, The First Hospital of Qiqihar, Qiqihar, P. R. China; b Pharmacy Department of the Second Affiliated Hospital of Qiqihar Medical College, Qiqihar, P. R. China; c First Department of Surgery, Gannan People’s Hospital, Qiqihar, P. R. China

**Keywords:** Gastric cancer, integrin-subunit alpha 11, PI3K, AKT, progression

## Abstract

Gastric cancer (GC) is among the most frequent malignancies originating from the digestive system worldwide, while the role and specific mechanism of integrin-subunit alpha 11 (ITGA11) in GC remain unclear. This study probes the expression characteristics and function of ITGA11 in GC. Firstly, the ITGA11 profile in GC tissues and paracancerous non-tumor tissues was assessed by quantitative reverse transcription-polymerase chain reaction (qRT-PCR) and Western blot (WB), and the association between ITGA11 and GC patients’ clinicopathological indicators was evaluated. ITGA11 knockdown models were set up in GC cell lines MKN45 and AGS. Cell proliferation was determined by the cell counting kit-8 (CCK-8) assay and colony formation assay. WB was utilized to gauge the expression of apoptosis-related proteins (Bax, Bcl2, Bad, and C-Caspase3) and the PI3K/AKT pathway. We discovered that the ITGA11 expression was boosted in GC tissues and was related to the unfavorable prognosis of GC patients. Additionally, ITGA11 knockdown abated GC cell proliferation, invasion and migration, and enhanced cell apoptosis. In animal experiments, the tumorigenesis of GC cells knocking down ITGA11 was reduced. Mechanically, knocking down ITGA11 notably inactivated the PI3K/AKT axis. The tumor-suppressive effect mediated by ITGA11 knockdown was attenuated after activating the PI3K/AKT pathway with insulin-like growth factor 1 (IGF-1). Overall, this study substantiated that the ITGA11 expression was heightened in GC tissues, which affected GC progression by modulating the PI3K/AKT pathway.

## 1. Introduction

Gastric cancer (GC), among the common malignancies originating from the digestive system, is the second risk factor of cancer-associated mortality and the fourth most common cancer globally [1,2]. With the development of clinical technology, great progress has been made in the diagnosis and staging, genome classification, surgical resection and treatment, systemic radiotherapy and chemotherapy, targeted therapy, and immunotherapy of peritoneal diseases [3]. However, due to the insidious onset and highly postoperative recurrence and metastasis rate, GC treatment is still challenging [4]. Therefore, it is crucial to find strategies for GC development and treatment.

Integrin acts as a bridge between the extracellular matrix (ECM) and the cytoskeleton, controlling cell proliferation, differentiation, tumor invasion, and metastasis [5]. Recently, the role of integrin in tumors has attracted extensive attention. The integrin family consists of 18 distinct α subunits and eight distinct β subunits, including integrin Subunit Alpha 11 (ITGA11), also known as recombinant integrin Alpha 11. It is reported that overexpressed ITGA11 is strongly linked to breast cancer suffers’ unfavorable prognosis [6]. In addition, Wu P and Ando T et al. claimed that ITGA11 is up-regulated in non-small cell lung cancer (NSCLC), and overexpressing ITGA11 facilitates tumor progression and postoperative recurrence [7,8]. These reports illustrate that ITGA11 is an underlying therapeutic molecule for various tumors. Nevertheless, ITGA11’s mechanism of action in GC has seldom been reported.

According to reports, the PI3K/AKT axis exerts a vital role in regulating cell proliferation and differentiation as a classical signaling pathway. Abnormal activation of PI3K/AKT substantially affects multiple tumors [9], and PI3K/AKT is considered a key pathway in the development of prostate cancer [10], endometrial cancer [11], and laryngeal cancer [12]. Additionally, PI3K/AKT contributes to GC. For example, casein kinase 2a1 (CSNK2A1) facilitates GC evolvement via PI3K/AKT/mTOR [13]. Similarly, Zhao H et al. indicated that knocking down CEACAM19 represses GC cell proliferation, migration, and invasion by blocking PI3K/AKT and NF-κB [14]. However, whether ITGA11 affects GC through PI3K/AKT remains to be further explored.

Previous studies suggest that ITGA11 functions as an oncogene in tumors. Therefore, we suppose that ITGA11 is also involved in GC progression. Here, we detected the ITGA11 expression in GC tissues and probed the relationship between ITGA11 and the PI3K/AKT pathway. The novel ITGA11/PI3K/AKT axis may exert a dominant role in GC evolvement and metastasis. These outcomes reveal a new molecular mechanism in GC evolvement and provide a reference value for the therapy and prognosis of GC.

## 2 Materials and Methods

### 2.1 Specimen collection and manipulations

The cancerous tissues of 49 GC patients who underwent gastrectomy in our hospital were obtained from March 2018 to March 2020. Before the operation, patients did not receive chemotherapy, radiotherapy, or other adjuvant treatment. The control samples were collected from the same patient’s adjacent non-tumor tissues (3 cm from the surgical margin at any rate), and no tumor cells were observed on postoperative pathological examination. The diagnosis of GC was confirmed following World Health Organization (WHO) criteria. All specimens were removed and preserved in liquid nitrogen at −196°C until adopted for RNA separation. Our research was granted by the research ethics committee of our hospital and received informed consent from all participating patients.

### 2.2 Cell culture and manipulation

GC cell lines MKN45 and AGS were ordered from the Cell Center of the Chinese Academy of Sciences (Shanghai, China). They were cultured with RPMI1640 comprising 10% fetal bovine serum (FBS) and 1% penicillin/streptomycin (Invitrogen, CA, USA) in an incubator at 37°C with 5% CO_2_. RPMI1640 and FBS were afforded by Thermo Fisher Scientific (MA, USA). Cells were trypsinized and sub-cultured with 0.25% trypsin (Thermo Fisher HyClone, Utah, USA) during the logarithmic growth phase.

### 2.3 Cell transfection and treatment

siRNA against ITGA11 (Si-ITGA11#1, Si-ITGA11#2, Si-ITGA11 #3) and the corresponding negative control (Si-NC) were afforded by Geneharma (Shanghai, China). The PI3K/AKT agonist IGF-1 was obtained from MedChemExpress (Cat.No. HY-P70788, Shanghai, China) and adopted for activating the PI3K/AKT pathway at a dose of 50 ng/mL [15]. MKN45 and AGS cells were seeded on 24-well plates (2 × 10^4^ cells/well), kept at 37°C with 5% CO_2_ for 24 hours, and transfected with si-ITGA11 or si-NC along with Lipofectamine® 3000 (Invitrogen; ThermoFisherScientific, Inc.). Following 24 hours, the culture medium was discarded and refreshed with a complete one. After another 24-hour culture, the transfection validity was measured by quantitative reverse transcription-polymerase chain reaction (qRT-PCR) and Western blot (WB) [16].

### 2.4 Cell counting kit-8 (CCK-8) assay

MKN45 and AGS cells were seeded on 24-well plates (2 × 10^4^ cells/well), incubated at 37°C with 5% CO_2_ for 24 hours, and transfected with si-ITGA11 or si-NC along with Lipofectamine® 3000 (Invitrogen; ThermoFisher Scientific, Inc.). Following 24 hours, the culture medium was refreshed with a complete one. After another 24-hour culture, the ITGA11 mRNA and protein level was gauged by qRT-PCR or WB for confirming stable transfection. For evaluating cell viability, MKN45 and AGS cells were inoculated into 96-well plates (1 × 10^3^ cells/well) and maintained for 24 hours. Then, 10 μL CCK-8 reagent (Dojindo Molecular Technologies, Kumamoto, Japan) was added to each well, following the manufacturer’s guidelines. After one hour of incubation at 37°C, the optical density (OD) value at 450 nm was reviewed on a spectrophotometer (Bio-Rad, CA, USA). Each test was implemented in triplicate.

### 2.5 Colony formation experiment

The transfected MKN45 and AGS cells were inoculated into 6-well plates (1000 cells/well). They were then kept in the RPMI-1640 medium (contained 10% FBS and 1% penicillin/streptomycin) for 2 weeks. Afterward, the cells underwent immobilization with formaldehyde (10 min) and staining with 0.5% crystal violet (10 min). After being rinsed with PBS buffer, the stained colonies were imaged and calculated using a light microscope (Bx23, Olympus) [17]. Each test was conducted three times.

### 2.6 Transwell assay

After dispersion with 0.25% trypsin, MKN45 and AGS cells were centrifuged, resuspended, and seeded in individual wells of 24-well plates. The 8 µM pore size Matrigel Chambers (Corning, Beijing, China) were employed for the invasion assay but not for the migration test. The upper chamber was supplemented with 5 × 10^4^ transfected cells, and Matrigel matrix gel was added. In parallel, the medium comprising 10% FBS was put in the lower chamber, which was filled with 400 μL of RPMI-1640. Following incubation at 37°C for 24 hours, the cells that failed to migrate were cleared. Transwell membranes were secured with 4% paraformaldehyde for 10 min and then stained with 0.5% crystalline violet. After being flushed with tap water, the cells were calculated under an inverted microscope. All tests were conducted three times.

### 2.7 Tissues immunofluorescence

Paraffin sections underwent dewaxing and hydration, antigen repair, blocking and incubation with the primary Anti-p-PI3K antibody (ab182651) and Anti-p-AKT antibody (ab18785). Next, they were maintained with secondary antibodies, maintained with 4,6 diamidino-2-phenylindole (DAPI), nucleated and photographed on a fluorescence microscope (BX53, Olympus, China) [18]. Three sections of each specimen were taken, and five non-overlapping high-powered fields of view were chosen at random for each section.

### 2.8 Terminal deoxynucleotidyl transferase-mediated dUTP-biotin nick end labeling assay (TUNEL) staining

MKN45 and AGS cells were centrifuged, resuspended, and seeded in individual wells of 24-well plates (each well contained 2 × 10^5^ cells). 24 hours later, the culture medium was discarded, and the cells were fixed in 4% paraformaldehyde at room temperature at room temperature for 30 min. Next, the cells were staining using TUNEL staining solution (Beyotime, Shanghai, China) for 1 hour in 37°C. The nucleus was staining DAPI (Beyotime, Shanghai, China) as per the manufacturer’s directions.

To evaluate apoptosis *in vivo*, all samples were fastened in 4% paraformaldehyde overnight at 4°C, permeabilized with 0.1% Triton X-100 and then maintained with the TUNEL staining solution (Beyotime, Shanghai, China) for 1 hour as per the manufacturer’s directions [19]. TUNEL-positive cells were counted in five randomly chosen areas of the slide under a microscope (Olympus).

### 2.9 qRT-PCR

Total cellular RNA was separated by adopting the Trizol reagent and then transcribed into cDNA with the PrimeScript™ RT Reagent kit (Invitrogen, Shanghai, China). The qPCR was implemented using the Bio-Rad CFX96 quantitative PCR system and SYBR, with initial denaturation (95°C, 5 min), denaturation (95°C, 15 s) and annealing (60°C, 30 s). β-actin was the housekeeping gene of ITGA11, with the 2 ^(-ΔΔCt)^ method for statistics. Each test was performed in triplicate. All primers were devised and synthesized by Guangzhou Ribo Biotechnology Co. Ltd. Primer sequences are shown in Table 2.

### 2.10 Western blot (WB)

After cells handling, the culture medium was removed, and protein lysate (Roche) was added to isolate the total protein. Following gel electrophoresis, 50 µg total protein was transferred to polyvinylidene fluoride (PVDF) membranes. The membranes were then sealed with 5% skimmed milk at room temperature (RT) for one hour, cleared with TBST three times (10 min each), and incubated with the primary antibodies (1:1000; Abcam, MA, USA) of Bcl-2 (ab32124), Bax (ab32503), p-PI3K (ab182651), PI3K (ab154598), p-AKT (ab18785), AKT (Ab131168), ITGA11 (ab198826), and β-actin (ab115777) overnight at 4°C. After being flushed with TBST, the membranes were kept at RT with horseradish peroxidase (HRP)-tagged anti-rabbit secondary antibody (1:300) for 1 hour. Subsequently, they were rinsed with TBST three times (10 min each). Finally, the Western blot reagent (Invitrogen) was adopted for color imaging, and the gray intensity was analyzed by Image J.

### 2.11 Xenograft tumor experiment

Sixty BALB/c-nu nude mice (4 to 6 weeks old, ordered from the Animal Center of Harbin Medical University) were selected to establish an *in-vivo* xenograft tumor model. MKN45 and AGS cells knocking down ITGA11 were adjusted to reach a density of 2 × 10^7^ mL^−1^. Then, 0.1 mL cell suspension was administered subcutaneously into the left forelimb armpit of each mouse. Within 30 days following the administration, the mice’s livability, body weight and survival conditions were recorded. Tumor volume (*V*) = 0.5 × a (the longest diameter of the tumor) ×b (the shortest diameter perpendicular to the long diameter)^2^ [20]. After continuous drug administration, mice were dislocated and killed on the 30th day. Finally, the tumor was stripped off and weighed. All animal tests were authorized by the Ethics Committee of the First Hospital of Qiqihar.

### 2.12 Data Process

The SPSS22.0 statistical software (SPSS Inc., Chicago, IL, USA) was adopted for data analysis. Data conforming to normal distribution were presented as mean ± standard deviation (x ± s). Two groups of data were compared by *t* test. The comparison between multiple groups was made by one-way ANOVA, and the LSD-t method was employed for multiple comparisons between groups. When *P* < 0.05, the statistics were significant.

## 3. Results

### 3.1 Expression of ITGA11 in gastric cancer tissues

**Figure 1.**
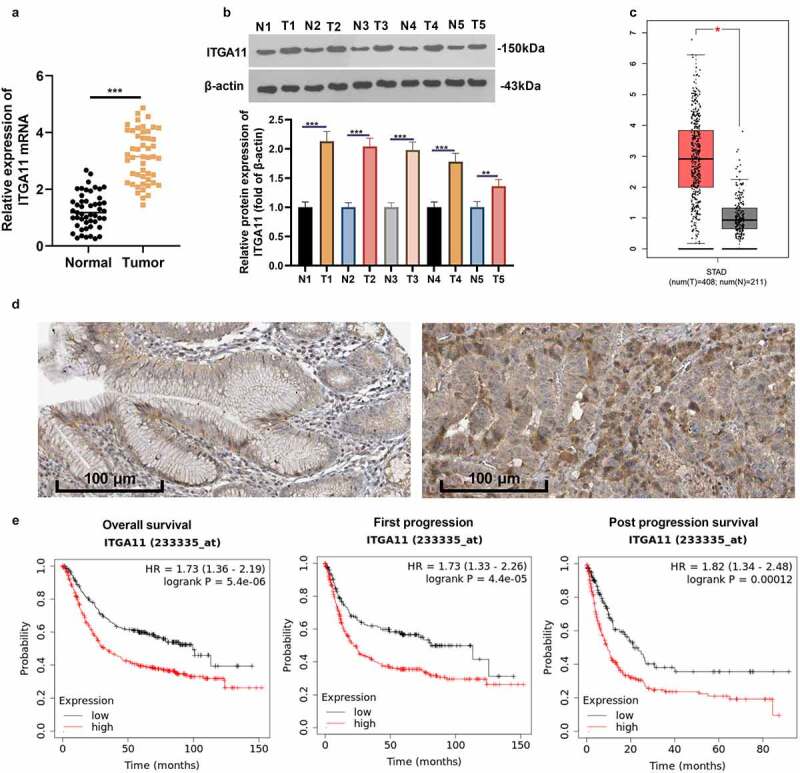
Expression of ITGA11 in GC

We analyzed the ITGA11 expression in 49 clinical samples to probe the expression characteristics of ITGA11 in GC tissues. As a result, ITGA11 was highly expressed in GC tissues versus non-tumor tissues (*P* < 0.05, Figure 1(a)). As substantiated by WB outcomes, the ITGA11 profile was obviously uplifted in GC tissues versus normal tissues (*P* < 0.05, Figure 1(b)). By querying the GEPIA database (http://gepia.cancer-pku.cn/) and The Human Protein Atlas (https://www.proteinatlas.org/), we discovered that the ITGA11 profile in GC tissues was overtly higher than that in paracancerous non-tumor tissues (Figure 1(c-d)). More importantly, patients with high ITGA11 expression had larger tumor volume, later clinical staging, and more distant metastasis and vascular invasion (vs. patients with lower ITGA11 expression, Table 1). Meanwhile, the data from the Kaplan–Meier Plotter database (http://kmplot.com/analysis/) corroborated that the higher ITGA11 expression was linked to poorer overall survival, first progression and post-progression survival of GC sufferers (Figure 1(e)). Thus, ITGA11 was a potential carcinogenic gene in GC and was an unfavorable predictor of GC patients.

**Table 1.** Relationship between the ITGA11 expression in GC tissue samples and clinical characteristics

Characteristics	Patients	Expression of ITGA11		P-value
		High- ITGA11	Low-ITGA11	
Total	49	27	22	
**Age(years)**				0.897
<63	25	14	11	
≥63	24	13	11	
**Gender**				0.335
Male	26	16	10	
Female	23	11	12	
**Tumor location**				0.659
Bottom	21	13	8	
Body	15	8	7	
Gastric antrum	13	6	7	
**Diameter**				0.013*
<3 cm	26	10	16	
≥3 cm	23	17	6	
**Clinical stage**				0.030*
Early	24	17	7	
Middle and late	25	10	15	
**Distant metastasis**				0.030*
Without	25	10	15	
With	24	17	7	
**Vascular invasion**				0.038*
Yes	28	19	9	
No	21	8	13	

(Note: *P < 0.05 was statistically significant)

**Table 2.** Primer sequences

Gene name	Primer sequences (5`→3`)
ITGA11	forward: GCAGTGACAGTAATGAGCGG reverse: TGAAGATGCAGCTGAAGGGA
β-actin	forward: GGCATCCTCACCCTGAAGTA reverse: GAAGGTGTGGTGCCAGATTT

A: The ITGA11 profile in cancerous and paracancerous normal tissues of 49 clinical samples was examined by qRT-PCR. B: The ITGA11 profile in five cases of cancerous and paracancerous GC tissues was verified by WB. C: The GEPIA database was adopted to verify ITGA11 expression in GC tissue. D: The effect of ITGA11 on the GC prognosis was analyzed in the human protein altas (https://www.proteinatlas.org/). E. Kaplan-Meier Plotter database (http://kmplot.com/analysis/) was adopted for analyzing the association of ITGA11 levels with overall survival, first progression and post progression survival of GC sufferers. **P < 0.01, ***P < 0.001

### 3.2 Inhibiting ITGA11 attenuated gastric cancer cell proliferation and invasion and facilitated apoptosis

**Figure 2.**
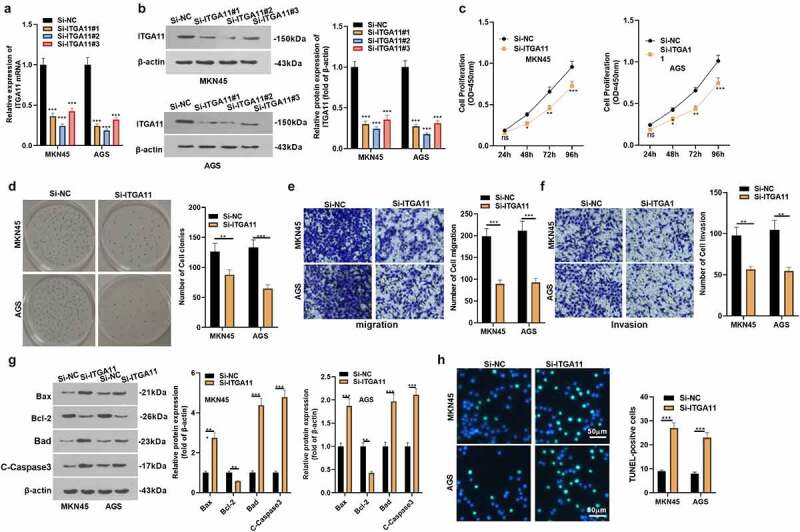
Inhibiting ITGA11 attenuated GC cell proliferation and invasion and strengthened apoptosis

To explore the effect of ITGA11 on the evolvement of GC, we constructed an ITGA11 knockdown model in MKN45 and AGS and confirmed the transfection validity by qRT-PCR and WB to figure out the influence of ITGA11 on GC progression (*P* < 0.05, Figure 2(a-b)). The CCK-8 assay and colony formation test disclosed MKN45 and AGS cell viability declined after ITGA11 knockdown (*P* < 0.05, Figure 2(c-d)). Transwell assay manifested that attenuating ITGA11 weakened the migrative and invasive abilities of MKN45 and AGS (*P* < 0.05, Figure 2(e-f)). We applied WB to examine the impact of ITGA11 knockdown on cell apoptosis and discovered that ITGA11 knockdown impeded the anti-apoptotic protein Bcl-2 level but up-regulated the pro-apoptotic protein Bax, Bad and C-Caspase3 (*P* < 0.05, Figure 2(g)). At the same time, TUNEL outcomes displayed that by contrast with the control group, the positive cell number of ITGA11 was distinctly augmented by knocking down ITGA11(*P* < 0.05, Figure 2(h)). In conclusion, hindering ITGA11 attenuated the malignant behaviors of MKN45 and AGS.

ITGA11 knockdown models were constructed in MKN45 and AGS cells. A-B: qRT-PCR and WB were implemented to test the effect of ITGA11 knockdown plasmids. C-D: Cell proliferation was verified by CCK-8 and colony formation experiment. E-F: The migrative and invasive ability of MKN45 and AGS were monitored by Transwell assay. G: The profiles of Bcl-2, Bad, C-Caspase3 and Bax were monitored by WB. H: TUNEL assay was adopted to gauge the apoptosis of MKN45 and AGS cells. Ns *P* > 0.05, *P < 0.05, **P < 0.01, ***P < 0.001. N = 3

### 3.3 The regulatory effect of ITGA11 on the ITGA11/ PI3K/AKT pathway

**Figure 3.**
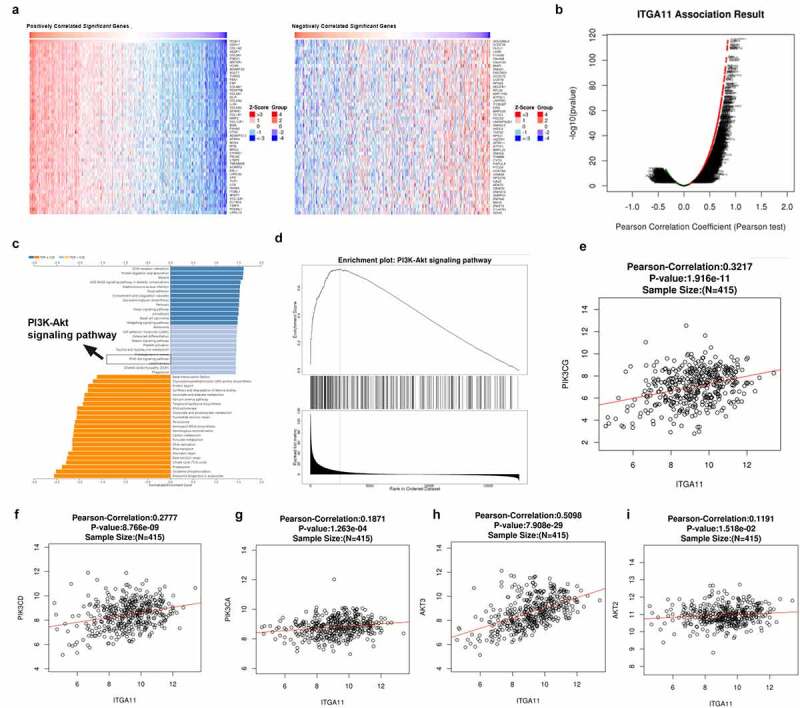
The regulatory effect of ITGA11 on the ITGA11/ PI3K/AKT pathway

In order to investigate the downstream mechanism of ITGA11 in GC, we analyzed the co-expressed genes of ITGA11 in GC with the LinkedOmics database (http://linkedomics.org/login.php). The Top-50 positively co-expressed genes and the Top-50 negatively co-expressed genes of ITGA11 in GC were shown (Figure 3(a)). The overall closely co-expressed genes of ITGA11 in GC were exhibited as a volcano plot (Figure 3(b)). Through LinkedOmics database, we performed Gene Set Enrichment Analysis (GSEA) using those co-expressed genes in LUAD. We observed that the PI3K/AKT pathway was associated with ITGA11 (p < 0.05, Figure 3(c-d)). Moreover, we found that ITGA11 has positive relationships with PIK3CG, PIK3CD, PIK3CA, AKT2, and AKT3 (Figure 3(e-i)). These data hinted that ITGA11 is significantly associated with the PI3K/AKT pathway in GC.

LinkedOmics database (http://linkedomics.org/login.php) was used for analyzing the potential downstream targets of ITGA11. A: The positively and negatively co-expressed Top-50 genes of ITGA11 in GC were displayed in heat maps. B: The overall closely co-expressed genes of ITGA11 in GC were exhibited as a volcano plot. C-D: PI3K-AKT pathway has a potential role in ITGA11 in GC. The data was analyzed by GSEA. E-I: The LinkedOmics database analyzed the correlation between ITGA11 and PIK3CG, PIK3CD, PIK3CA, AKT2, and AKT3

### 3.4 Inhibiting ITGA11 blocked the PI3K/AKT pathway

**Figure 4.**
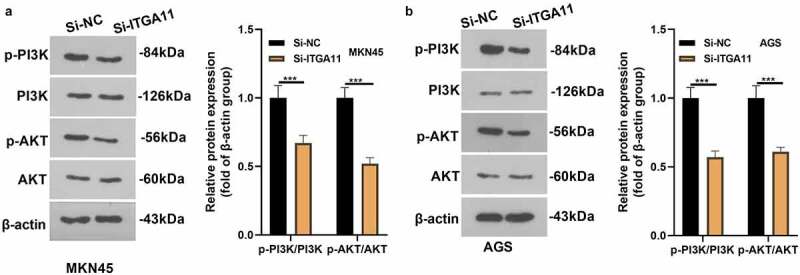
Inhibiting ITGA11 inactivated PI3K/AKT

To confirm whether PI3K/AKT pathway involves in ITGA11-mediated GC progression, the PI3K/AKT level in MKN45 and AGS was tested by WB. As a result, the PI3K/AKT profile was hindered after the ITGA11 knockdown (*P* < 0.05, Figure 4(a-b)), manifesting that inhibiting ITGA11 choked the PI3K/AKT pathway.

ITGA11 knockdown models were established in MKN45 and AGS. A-B. WB was applied to make certain the PI3K/AKT profile in MKN45 and AGS cells. * * * *P* < 0.001, N = 3

### 3.5 Activating PI3K abated the effect of ITGA11 knockdown in gastric cancer progression

**Figure 5.**
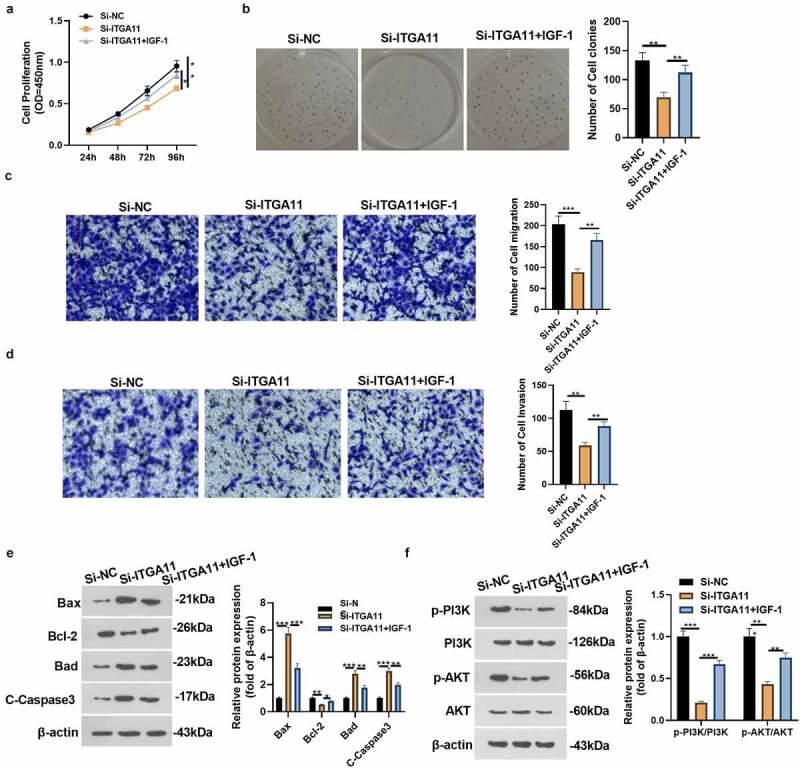
Activating PI3K repressed the effect of ITGA11 knockdown on GC progression

For the aim of confirming PI3K pathway activation can reversed the anti-tumor role-mediated by si-ITGA11, we added the ITGA11 knockdown plasmid and/or PI3K/AKT agonist IGF-1 into MKN45 to explore the specific mechanism of ITGA11 in GC progression. The CCK-8 assay and colony formation test were adopted to verify cell proliferation. It was discovered that cell proliferation was reduced after knocking down ITGA11 (vs. the Si-NC group). However, IGF-1 intervention facilitated cell proliferation versus the Si-ITGA11 group (*P* < 0.05, Figure 5(a-b)). Transwell assay manifested that MKN45 cell migration and invasion were dampened after ITGA11 knockdown (vs. the Si-NC group). Nonetheless, IGF-1 intervention strengthened cell migration and invasion versus the Si-ITGA11 group (*P* < 0.05, Figure 5(c-d)). Moreover, WB substantiated that knocking down ITGA11 suppressed the Bcl-2 profile and up-regulated Bax, Bad and C-Caspase3. In contrast, the results were completely opposite after IGF-1 interventions (*P* < 0.05, Figure 5(e)). Meanwhile, knockdown of ITGA11 caused inhibition of PI3K/AKT phosphorylation levels (vs. the Si-NC group), whereas PI3K/AKT phosphorylation levels were augmented after IGF-1 application (vs. the Si-ITGA11 group) (*P* < 0.05, figure 5(f)). These conclusions corroborated that attenuating ITGA11 exerted anti-tumor effects by restraining PI3K/AKT in GC cells.

ITGA11 knockdown plasmids and/or IGF-1 were added to MKN45 cells. A-B: The CCK-8 assay and colony formation test were carried out to gauge cell proliferation. C-D: Cell migration and invasion were assessed by Transwell assay. E: The profiles of Bcl-2 and Bax were monitored by WB. F: WB gauged the protein levels of the PI3K/AKT axis in MKN45 cells. * *P* < 0.05, * * *P* < 0.01, * * * *P* < 0.001, N = 3

### 3.6 *Knocking down ITGA11 repressed gastric cancer cell growth* in vivo

**Figure 6.**
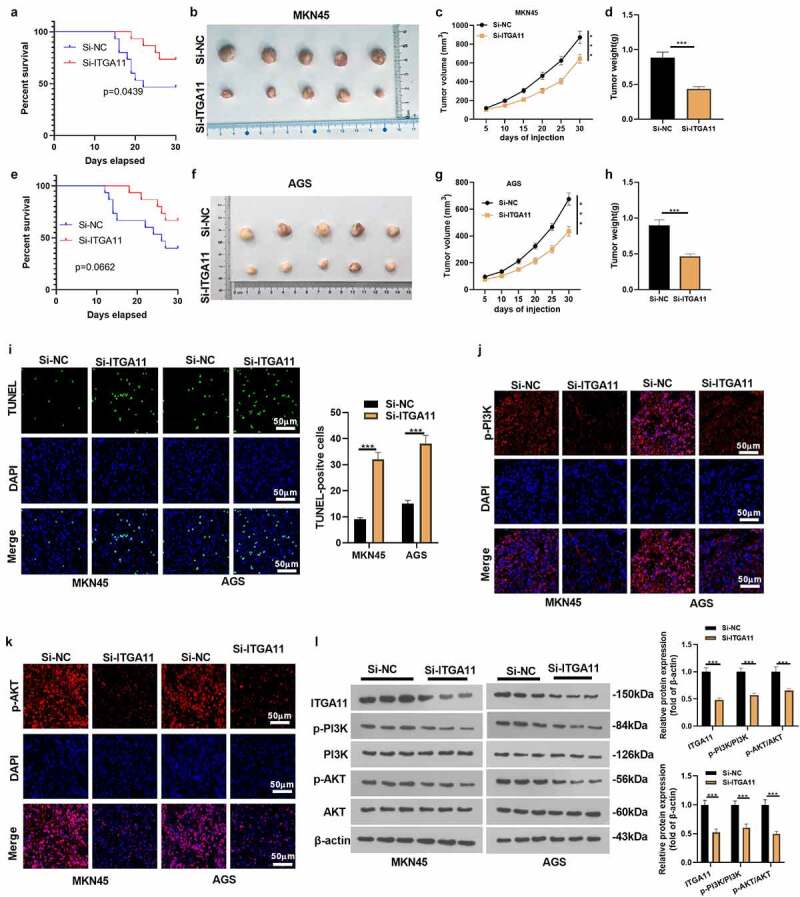
Knocking down ITGA11 dampened GC cell growth cells *in vivo.*

We constructed ITGA11 knockdown tumor models in MKN45 and AGS to clarify the influence of ITGA11 on tumor growth and metastasis *in vivo*. An analysis of the survival rate of mice displayed that knockdown of ITGA11 improved the mice’s survival rate (Figure 6(a), e). Knocking down ITGA11 choked tumor volume and weight (*P* < 0.05, Figure 6(b-h)). As manifested by TUNEL data, knockdown of ITGA11 resulted in distinct facilitation in TUNEL-positive cell number versus the control group (*P* < 0.05, Figure 6(i)). In parallel, tissue immunofluorescence outcomes uncovered that knockdown of ITGA11 brought about notably declined fluorescence intensity of PI3K/AKT versus the control group (*P* < 0.05, Figure 6(j-k)). Besides, WB established that si-ITGA11 down-regulated ITGA11 and repressed PI3K/AKT pathway activation (*P* < 0.05, Figure 6(i)), further confirming that abating ITGA11 repressed GC growth *in vivo*.

Mouse tumor models knocking down ITGA11 were constructed in MKN45 and AGS cell lines. A: Analysis of survival rates in mice. B-G: After 30 days, the nude mice were sacrificed, and their subcutaneous nodules were removed, with tumor volume and weight calculated. H: The TUNEL assay was employed to examine apoptosis in tumor tissues. I: The fluorescence intensity of the PI3K/AKT pathway was checked by tissue immunofluorescence. J: The profiles of ITGA11 and PI3K/AKT were tested by WB. * * * *P* < 0.001, N = 5

## 4. Discussion

GC is the second risk factor of tumor-associated mortality in China, second only to lung cancer. It is a multi-factorial and highly invasive disease with stages [21]. However, its molecular mechanisms are not fully understood. Therefore, the exploration of GC pathogenesis and the search for treatment are helpful in improving GC prognosis. Here, we found that ITGA11 facilitated GC cell proliferation and metastasis and inhibited apoptosis. Further experiments demonstrated that inhibiting ITGA11 weakened the PI3K/AKT activation, contributing to developing new therapeutic targets for GC.

ITGA11 is a novel collagen-binding integrin, which acts as a mesenchymal collagen receptor and mediates the migration and collagen recombination of mesenchymal non-muscle cells [22]. Studies have shown that ITGA11 contributes to regulating fibroblast differentiation in diabetic cardiomyopathy [23], mouse embryo implantation [24], atherosclerosis [25], and osteoarthritis [26]. More importantly, ITGA11 has been widely reported to have significant values in glioblastoma [27], head and neck squamous cell carcinoma [28], and breast cancer [29]. However, the biological role of ITGA11 in GC remains unclear. Here, we probed the impact of ITGA11 on GC. The results revealed that human GC cell lines and tissues exhibited enhanced expression levels of ITGA11. The database showed that ITGA11 was a poor prognostic factor in GC patients. Meanwhile, ITGA11 knockdown bridled GC cell proliferation, migration and invasion, and enhanced the apoptosis. Mechanism research also manifested that ITGA11 exerted its biological function through PI3K/Akt, suggesting that ITGA11 was a positive regulator of GC, which was confirmed *in vivo*.

The PI3K/AKT axis is a common pathway contributing to various cancers. Multiple reports have shown that PI3K/AKT exerts crucial functions in GC. For instance, Zhao et al. found that the deletion of PDZK1 expression in GC activated PI3K/AKT, leading to poor prognosis [30]. XLOC_006753 enhances the resistance of GC cells through PI3K/AKT [31]. At the same time, PI3K/AKT induces GC cells’ stemness [32]. Therefore, the PI3K/AKT pathway has attracted extensive attention as a potential therapeutic target. Although PI3K/AKT’s biological function in GC progression has been well-established, the function of ITGA11 in modulating PI3K/AKT remains unclear. This study manifested that ITGA11 regulated PI3K/AKT. Inhibiting ITGA11 inactivated the PI3K/AKT pathway. Further, we used IGF-1 to activate the PI3K/AKT pathway and discovered that IGF-1 elevated tumor growth and weakened the tumor-suppressive effect mediated by ITGA11 knockdown in GC. Hence, the PI3K/AKT activator can promote GC progression, which is consistent with previous reports [33,34].

## Conclusion

Overall, ITGA11 is involved in GC progression and plays a carcinogenic role in GC by activating PI3K/AKT. Inhibiting ITGA11 reduces GC cell proliferation, migration, and invasion and facilitates apoptosis by suppressing PI3K/AKT. These results provide valuable insights for GC treatment and prognosis. However, we have not confirmed the reliability of this mechanism *in vivo*, which will be further explored in subsequent experiments.

## Data Availability

The data sets used and analyzed during the current study are available from the corresponding author on reasonable request.

## List of abbreviations

Abbreviations: Full names
CCK-8: Cell counting kit-8
CSNK2A1: Casein kinase 2a1
ECM: Extracellular matrix
FBS: Fetal bovine serum
GC: Gastric cancer
GEPIA: Gene Expression Profiling and Interactive Analyses
GSEA: Gene Set Enrichment Analysis
HRP: Horseradish peroxidase
IGF-1: insulin like growth factor 1
ITGA11: Integrin subunit alpha 11
KEGG: Kyoto Encyclopedia of Genes and Genomes
NSCLC: Non-small cell lung cancer
OD: Optical density
PI3K: phosphatidylinositol 3-kinase
PVDF: Polyvinylidene fluoride
qRT-PCR: quantitative reverse transcription-polymerase chain reaction
RT: Room temperature
WB: Western blot
WHO: World Health Organization

## References

[1] Song Z, Wu Y, Yang J, et al. Progress in the treatment of advanced gastric cancer. Tumour Biol. 2017 Jul;39(7):1010428317714626. 28671042 10.1177/1010428317714626

[2] Ang TL, Fock KM. Clinical epidemiology of gastric cancer. Singapore Med J. 2014 Dec;55(12):621–628. 25630323 10.11622/smedj.2014174 PMC4291998

[3] Johnston FM, Beckman M. Updates on Management of Gastric Cancer. Curr Oncol Rep. 2019 Jun 24;21(8):67. 31236716 10.1007/s11912-019-0820-4

[4] Petryszyn P, Chapelle N, Matysiak-Budnik T. Gastric Cancer: where Are We Heading? Dig Dis. 2020;38(4):280–285. Epub 2020 Feb 17. 32062657 10.1159/000506509

[5] Bianconi D, Unseld M, Prager GW. Integrins in the Spotlight of Cancer. Int J Mol Sci. 2016 Dec 6;17(12):2037. 10.3390/ijms17122037 PMC5187837 27929432

[6] Pan Y, Liu G, Yuan Y, et al. Analysis of differential gene expression profile identifies novel biomarkers for breast cancer. Oncotarget. 2017 Dec 8;8(70):114613–114625. 29383106 10.18632/oncotarget.23061 PMC5777718

[7] Wu P, Wang Y, Wu Y, et al. Expression and prognostic analyses of ITGA11, ITGB4 and ITGB8 in human non-small cell lung cancer. PeerJ. 2019;7:e8299. 31875161 10.7717/peerj.8299 PMC6927340

[8] Ando T, Kage H, Matsumoto Y, et al. Integrin α11 in non-small cell lung cancer is associated with tumor progression and postoperative recurrence. Cancer Sci. 2020 Jan;111 2:200–208.Epub 2019 Dec 18 31778288 10.1111/cas.14257 PMC6942423

[9] Pompura SL, Dominguez-Villar M. The PI3K/AKT signaling pathway in regulatory T-cell development, stability, and function. J Leukoc Biol. 2018 Jan 22. 10.1002/JLB.2MIR0817-349R. Epub ahead of print. 29357116

[10] Shorning BY, Dass MS, Smalley MJ, et al. The PI3K-AKT-mTOR Pathway and Prostate Cancer: at the Crossroads of AR, MAPK, and WNT Signaling. Int J Mol Sci. 2020 Jun 25;21(12):4507. 10.3390/ijms21124507 PMC7350257 32630372

[11] Slomovitz BM, Coleman RL. The PI3K/AKT/mTOR pathway as a therapeutic target in endometrial cancer. Clin Cancer Res. 2012 Nov 1;18(21):5856–5864. Epub 2012 Oct 18. 23082003 10.1158/1078-0432.CCR-12-0662

[12] Jiang T, Zhou ML, Fan J. Inhibition of GLUT-1 expression and the PI3K/Akt pathway to enhance the chemosensitivity of laryngeal carcinoma cells in vitro. Onco Targets Ther. 2018 Nov 6;11: 7865–7872. 30464533 10.2147/OTT.S176818 PMC6228052

[13] Jiang C, Ma Z, Zhang G, et al. CSNK2A1 Promotes Gastric Cancer Invasion Through the PI3K-Akt-mTOR Signaling Pathway. Cancer Manag Res. 2019;11:10135–10143. 31819646 10.2147/CMAR.S222620 PMC6897054

[14] Zhao H, Xu J, Wang Y, et al. Knockdown of CEACAM19 suppresses human gastric cancer through inhibition of PI3K/Akt and NF-κB. Surg Oncol. 2018 Sep;27 3:495–502.Epub 2018 May 3 30217308 10.1016/j.suronc.2018.05.003

[15] Binsila BK, Selvaraju S, Ghosh SK, et al. EGF, GDNF, and IGF-1 influence the proliferation and stemness of ovine spermatogonial stem cells in vitro. J Assist Reprod Genet. 2020;37(10):2615–2630. 32821972 10.1007/s10815-020-01912-5 PMC7550450

[16] Zhu Q, Li Y, Li L, et al. MicroRNA-889-3p restrains the proliferation and epithelial-mesenchymal transformation of lung cancer cells via down-regulation of Homeodomain-interacting protein kinase 1 [published online ahead of print, 2021 Nov 1]. Bioengineered 2021. 10.1080/21655979.2021.2000283. PMC8810057 34723781

[17] Zhang N, Liu JF. MicroRNA (MiR)-301a-3p regulates the proliferation of esophageal squamous cells via targeting PTEN. Bioengineered. 2020;11(1):972–983. 32970954 10.1080/21655979.2020.1814658 PMC8291791

[18] Tang Y, Li Y, Xin D, et al. Icariin alleviates osteoarthritis by regulating autophagy of chondrocytes by mediating PI3K/AKT/mTOR signaling. Bioengineered. 2021;12(1):2984–2999. 34167449 10.1080/21655979.2021.1943602 PMC8806900

[19] Zhao J, Zhou K, Ma L, et al. MicroRNA-145 overexpression inhibits neuroblastoma tumorigenesis *in vitro* and *in vivo*. Bioengineered. 2020;11(1):219–228. 32083506 10.1080/21655979.2020.1729928 PMC7039631

[20] Ning L, Zhang M, Zhu Q, et al. miR-25-3p inhibition impairs tumorigenesis and invasion in gastric cancer cells *in vitro* and *in vivo*. Bioengineered. 2020;11(1):81–90. 31909687 10.1080/21655979.2019.1710924 PMC6961587

[21] Smyth EC, Nilsson M, Grabsch HI, et al. Gastric cancer. Lancet. 2020 Aug 29;396(10251):635–648. 32861308 10.1016/S0140-6736(20)31288-5

[22] Tiger CF, Fougerousse F, Grundström G, et al. alpha11beta1 integrin is a receptor for interstitial collagens involved in cell migration and collagen reorganization on mesenchymal nonmuscle cells. Dev Biol. 2001 Sep 1;237(1):116–129. 11518510 10.1006/dbio.2001.0363

[23] Talior-Volodarsky I, Connelly KA, Arora PD, et al. α11 integrin stimulates myofibroblast differentiation in diabetic cardiomyopathy. Cardiovasc Res. 2012 Nov 1;96(2):265–275.Epub 2012 Aug 6. 22869616 10.1093/cvr/cvs259

[24] Li Z, Jia J, Gou J, et al. Mmu-miR-126a-3p plays a role in murine embryo implantation by regulating Itga11. Reprod Biomed Online. 2015 Sep;31 3:384–393.Epub 2015 Jun 4 26194885 10.1016/j.rbmo.2015.05.016

[25] Mao Z, Wu F, Shan Y. Identification of key genes and miRNAs associated with carotid atherosclerosis based on mRNA-seq data. Medicine (Baltimore). 2018 Mar;97(13):e9832. 29595698 10.1097/MD.0000000000009832 PMC5895374

[26] Guo SM, Wang JX, Li J, et al. Identification of gene expression profiles and key genes in subchondral bone of osteoarthritis using weighted gene expression network analysis. J Cell Biochem. 2018 Sep;119 9:7687–7695.Epub 2018 Jun 15 29904957 10.1002/jcb.27118

[27] Shin J, Shim HG, Hwang T, et al. Restoration of miR-29b exerts anti-cancer effects on glioblastoma. Cancer Cell Int. 2017;17:104. 29176935 10.1186/s12935-017-0476-9 PMC5693545

[28] Ju JA, Godet I, Ye IC, et al. Hypoxia Selectively Enhances Integrin α5β1 Receptor Expression in Breast Cancer to Promote Metastasis. Mol Cancer Res. 2017 Jun;15 6:723–734.Epub 2017 Feb 17 28213554 10.1158/1541-7786.MCR-16-0338 PMC5510543

[29] Parajuli H, Teh MT, Abrahamsen S, et al. Integrin α11 is overexpressed by tumour stroma of head and neck squamous cell carcinoma and correlates positively with alpha smooth muscle actin expression. J Oral Pathol Med. 2017 Apr;46 4:267–275.Epub 2016 Oct 4 27699902 10.1111/jop.12493 PMC5396328

[30] Zhao C, Tao T, Yang L, et al. Loss of PDZK1 expression activates PI3K/AKT signaling via PTEN phosphorylation in gastric cancer. Cancer Lett. 2019 Jul 1;453:107–121. Epub 2019 Mar 29. 30930234 10.1016/j.canlet.2019.03.043

[31] Zeng L, Liao Q, Zou Z, et al. Long Non-Coding RNA XLOC_006753 Promotes the Development of Multidrug Resistance in Gastric Cancer Cells Through the PI3K/AKT/mTOR Signaling Pathway. Cell Physiol Biochem. 2018;51(3):1221–1236. Epub 2018 Nov 27. 30481766 10.1159/000495499

[32] Ni SJ, Zhao LQ, Wang XF, et al. CBX7 regulates stem cell-like properties of gastric cancer cells via p16 and AKT-NF-κB-miR-21 pathways. J Hematol Oncol. 2018 Feb 8;11(1):17. 29422082 10.1186/s13045-018-0562-z PMC5806263

[33] Wang G, Lu M, Yao Y, et al. Esculetin exerts antitumor effect on human gastric cancer cells through IGF-1/PI3K/Akt signaling pathway. Eur J Pharmacol. 2017 Nov 5;814:207–215. Epub 2017 Aug 25. 28847482 10.1016/j.ejphar.2017.08.025

[34] Wang J, Zhang Y, Dou Z, et al. Knockdown of STIL suppresses the progression of gastric cancer by down-regulating the IGF-1/PI3K/AKT pathway. J Cell Mol Med. 2019 Aug;23 8:5566–5575.Epub 2019 Jun 11 31187582 10.1111/jcmm.14440 PMC6653615

